# Suppression of mTOR pathway and induction of autophagy-dependent cell death by cabergoline

**DOI:** 10.18632/oncotarget.5744

**Published:** 2015-10-14

**Authors:** Shao Jian Lin, Zhi Gen Leng, Yu Hang Guo, Lin Cai, Yu Cai, Ning Li, Han Bing Shang, Wei-Dong Le, Wei Guo Zhao, Zhe Bao Wu

**Affiliations:** ^1^ Department of Neurosurgery, Ruijin Hospital, Shanghai Jiao Tong University School of Medicine, Shanghai 200025, China; ^2^ Department of Neurosurgery, First Affiliated Hospital of Wenzhou Medical University, Wenzhou 325000, China; ^3^ Institute of Health Sciences, Shanghai Institutes for Biological Sciences, Chinese Academy of Sciences-Shanghai Jiao Tong University School of Medicine, Shanghai 200025, China

**Keywords:** autophagy, cabergoline, prolactinoma, autophagic cell death, autophagic flux

## Abstract

Cabergoline (CAB), the first-line drug for treatment of prolactinomas, is effective in suppressing prolactin hypersecretion, reducing tumor size, and restoring gonadal function. However, mechanisms for CAB-mediated tumor shrinkage are largely unknown. Here we report a novel cytotoxic mechanism for CAB. CAB induced formation of autophagosome in rat pituitary tumor MMQ and GH3 cells at the early stage through inhibiting mTOR pathway, resulting in higher conversion rates of LC3-I to LC3-II, GFP-LC3 aggregation, and increased autophagosome formation. Interestingly, CAB treatment augmented lysosome acidification and resulted in impaired proteolytic degradation within autolysosomes. This blocked the autophagic flux, leading to the accumulation of p62 aggregation and undigested autolysosomes. Knockdown of ATG7, ATG5, or Becn1, could significantly rescue the CAB-mediated cell death of MMQ cells (*p* < 0.05). CAB-induced autophagy and blockade of autophagy flux participated in antitumoral action *in vivo*. In conclusion, our study provides evidence that CAB concomitantly induces autophagy and inhibits the autophagic flux, leading to autophagy-dependent cell death. These findings elucidate novel mechanisms for CAB action.

## INTRODUCTION

Prolactinomas are the most prevalent neuroendocrine tumors, accounting for approximately 40% of all pituitary adenomas [1]. Dopamine agonists (DAs), including bromocriptine (BRC) and cabergoline (CAB), are the first-line treatment for these adenomas [1–6]. They effectively suppress prolactin (PRL) hypersecretion, reduce tumor size, and restore gonadal function [4, 7]. CAB is longer acting, more effective, better tolerated, than BRC, with less side-effects [8]. CAB is also effective in patients resistant or poorly responsive to BRC [8]. Furthermore, CAB has been used in treating patients with other types of pituitary tumors, including acromaglic, clinically nonfunctioning, and ACTH-secreting adenomas [9], making it a valuable therapeutic drug for pituitary adenomas.

A large body of evidence indicates that DA selectively activates cell surface dopamine 2 receptors (D2R), leading to the suppressed transcription and expression of the *PRL* gene as well as to decreased synthesis and secretion of PRL [1, 2, 10]. In addition, DA, BRC, and CAB activate the short isoform of D2R (D2S) and induce apoptosis [11–14]. We showed that transfection with D2S expressing adenovirus sensitizes GH3 xenografts to BRC treatment in nude mice, as evidenced by increase in apoptosis with an activation of caspase-3 [15]. CAB-induced apoptosis may result from caspase activation through ERK, JNK, and p38MAPK pathways [11, 14–16]. However, other mechanisms may also be involved in CAB-mediated tumor shrinkage, in addition to apoptosis [2].

Crinophagy was the earliest description of pituitary autophagy, as reported by Christian de Duve in 1969 [17, 18]. Macroautophagy (called “authophagy” throughout this paper) involves the sequestration of cytoplasm by double-layered membranes to form autophagosomes, which fuse with lysosomes, in which their contents are degraded [19–21]. Autophagy serves as a cytoprotective mechanism in response to stress. In addition, autophagy can lead to cell death under specific circumstances, a process known as ‘autophagic cell death’ (ACD), which is distinguished from the other form of programmed cell death, i.e. apoptosis [22]. Therefore, ACD is considered as an alternative cell death mechanism, which is morphologically defined (especially by transmission electron microscopy, TEM) as a type of cell death that occurs in the absence of chromatin condensation but is accompanied by large-scale autophagic vacuolization of the cytoplasm [23]. The transition from protective autophagy to cytotoxic autophagy relies on a balance between autophagosome production and appropriate lysosomal degradation.

In this study, we provide evidence that CAB concomitantly induces autophagosome formation and inhibits the autophagic flux, leading to accumulation of undigested autophagosomes and/or autolysosomes that ultimately result in ACD. These findings elucidate novel mechanisms for CAB action, suggesting that it may be potentially used in medical management of other tumors in addition to pituitary adenomas.

## RESULTS

### CAB induces both apoptotic and non-apoptotic cell death

**Figure 1:**
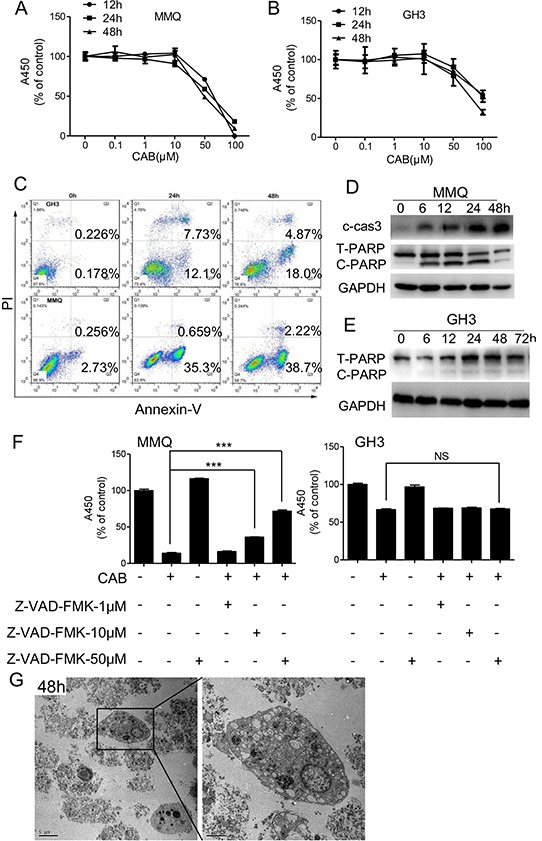
CAB induces both apoptosis and non-apoptosis cell death **A–B.** Cell survival was determined by the MTS assay. **C.** MMQ and GH3 cells were treated with CAB as indicated for apoptosis assay by Annexin V-FTIC and PI double staining. **D–E.** MMQ and GH3 cells were treated with CAB as indicated, and total proteins analyzed by Western blot using antibodies against caspase-3, PARP and GAPDH. **F.** MMQ and GH3 cells were treated with CAB in the presence or absence of Z-VAD-FMK at different concentrations for 48 hours, and cell survival was determined by the MTS. **G.** Transmission electron microscopy (TEM) images with enlargements; boxes showed autophagic vacuoles in MMQ cells treated with CAB for 48 h, and a cell death morphology accompanied by large-scale autophagic vacuoles in the cytoplasm with the absence of chromatin condensation.

To test for cell death induced by CAB, MTS assays were used to analyze in GH3 and MMQ cell lines. CAB decreased viability of GH3 and MMQ cells in both a dose- and time-dependent manner. Treatment with 50 μM CAB in MMQ cells for 48 h induced cell death by up to 50% (Fig. 1A); however, in GH3 cells, 100 μM CAB was required to produce a similar effect (Fig. 1B).

Previous studies have demonstrated that D2R agonists, such as CAB and BRC, induce apoptosis in pituitary tumors [12–14, 16, 24]. In accordance with those observations, apoptosis assay using PI and Annexin V-FITC double staining further revealed that CAB indeed rendered MMQ and GH3 cells to undergo apoptosis (Fig. 1C). CAB increased apoptotic related proteins such as cleaved caspase-3 and PARP, and induced caspase-dependent apoptosis in MMQ cells (Fig. 1D). However, in GH3 cells, CAB can induce cell death without PARP protein induction (Fig. 1E).

To characterize the CAB-induced cell death by apoptosis, we used Z-VAD-FMK, a pan caspase inhibitor, to treat the cells. In MMQ cells, Z-VAD-FMK can only partially block CAB-induced cell death in a dose-dependent manner (Fig. 1F). Furthermore, in GH3 cells, Z-VAD-FMK virtually failed to rescue cells from CAB-induced cell death (Fig. 1F). These findings suggest that CAB induce both apoptosis and non-apoptotic cell death. Therefore, MMQ cells were treated with CAB for 6, 12, 24 and 48 h, and were examined by transmission electron microscope (TEM). We noticed that, as early as 6 h of CAB exposure, large-scale autophagic vacuoles occurred in the cytoplasm (Fig. 1G and Supplemental Fig. 1A). At 12 h, cell death occurred and reached the peak after 48 h CAB treatment, in the absence of chromatin condensation but accompanied by large-scale autophagic vacuoles of the cytoplasm (Fig. 1G and Supplemental Fig. 1A).

Together, these results indicate that CAB mediates non-apoptotic cell death.

### CAB suppresses mTOR pathway

**Figure 2:**
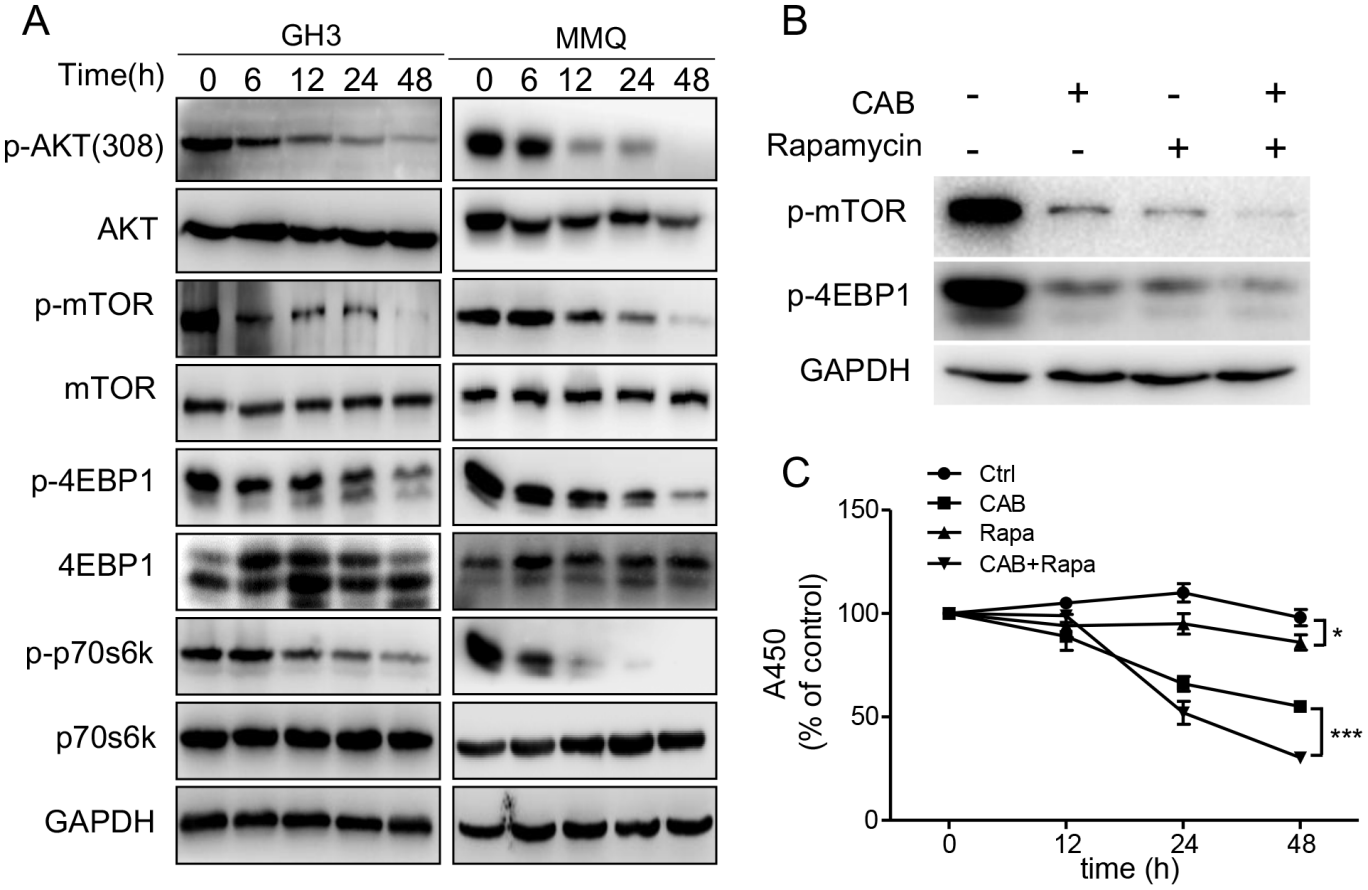
CAB suppresses the AKT/mTOR pathway **A.** CAB-treated GH3 and MMQ cellular lysates were blotted with antibodies recognizing p-mTOR (Ser 2448), mTOR, p-4EBP1 (Thr37/46), 4EBP1, p-p70s6k(Thr389) and p70s6k. A mouse anti-GAPDH antibody was utilized as a loading control. Blots are representative of three independent experiments. **B.** Immunoblots analysis of p-mTOR, p-4EBP1 and GAPDH in MMQ cells treated CAB with or without rapamycin. **C.** MMQ cells were treated with CAB in the presence or absence of rapamycin (100 nM) for 24 hours, and cell survival was determined by the MTS.

The phosphatidylinositol-3-kinase (PI3K)/Akt/mTOR pathway integrates signals from growth factors, nutrients and stresses to regulate cell survival and death [25]. We thus tested whether CAB could also inactivate AKT/mTOR pathway in pituitary cells. We found that AKT/mTOR signaling was significantly inhibited in the MMQ and GH3 cells by CAB in a time-dependent manner as indicated by decreased phosphorylation of AKT and mTOR as well as decreased phosphorylation of p70S6K and 4EBP1, two key downstream effectors of the mTOR pathway (Fig. 2A). On the other hand, rapamycin, an inhibitor of mTOR, combined with CAB treatment, further inhibited the phosphorylation of mTOR and 4EBP1 (Fig. 2B), and increased MMQ cell death (Fig. 2C).

### CAB induces autophagy

**Figure 3:**
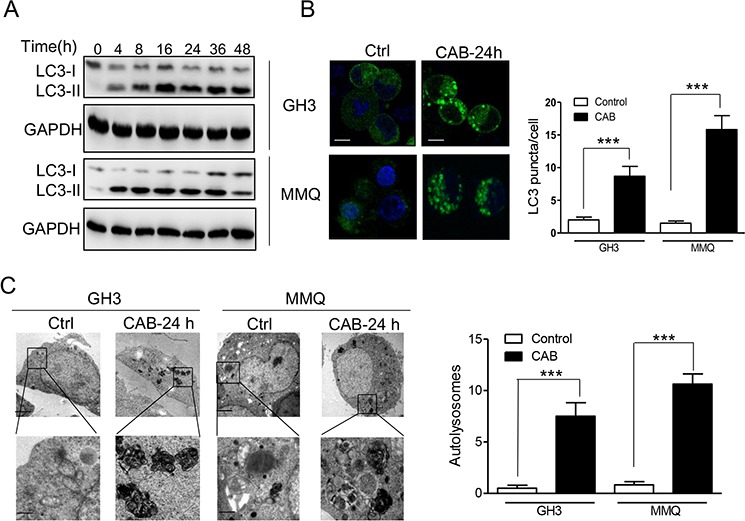
CAB induces autophagy **A.** Immunoblot analysis of LC3-I and LC3-II in GH3 and MMQ cells with or without CAB treatment at different time points. **B.** CAB treatment at 24 hours induced punctuative distribution of membrane-associated lipidated LC3-II in GH3-GFP-LC3 (left panels) and MMQ-GFP-LC3 (right panels) cells, observed with a confocal microscope. Scale bar represents 10 μm. Histogram shows the LC3 puncta from multiple experiments counting a total of 50 cells (mean ± SD). **C.** Electron micrographs of GH3 and MMQ cells with or without 24 h CAB treatment. Enlarged images of CAB treated cell (bottom parts) indicate autolysosomes (right). Histogram shows the autolysosome structures from multiple experiments counting a total of 50 cells (mean ± SD).

Having established that CAB suppressed AKT/mTOR pathway in GH3 and MMQ cells, we next investigated whether CAB also triggered autophagy, a novel cellular response to regulate cell fate on mTOR inactivation. During autophagy activation, microtubule-associated protein light chain 3-I (LC3-I) is converted to lipidated LC3-II, which is associated with autophagic vesicles and displays classical punctate distribution. This LC3-I to LC3-II conversion is a classical hallmark of autophagy [26]. As shown in Fig. 3A, CAB induced LC3-I conversion to LC3-II in MMQ and GH3 cells at early stages of CAB treatment; furthermore, in GFP-LC3-transfected MMQ and GH3 cells, CAB treatment induced the formation of LC3 puncta, whereas in the vehicle-treated control cells LC3-associated green fluorescence was diffused in cytoplasm (GH3: 8.7 ± 1.5 vs 2.0 ± 0.4, *n* = 50, *p* = 0.008; MMQ: 15.8 ± 2.1 vs 1.5± 0.3, *n* = 50, *p* < 0.001, respectively, Fig. 3B). TEM revealed increased numbers of autophagosomes and/or autolysosomes in the GH3 and MMQ cells treated with CAB compared with those in control cells (GH3: 7.5 ± 1.3 vs. 0.5 ± 0.3, *n* = 50, *p* = 0.002; MMQ: 10.6 ± 0.9 vs. 0.8 ± 0.3, *n* = 50, *p* < 0.0001; Fig. 3C), suggesting that CAB strongly induces autophagy activity in these cells.

### CAB blocks autophagic flux by impairing lysosomal degradation within autolysosomes

**Figure 4:**
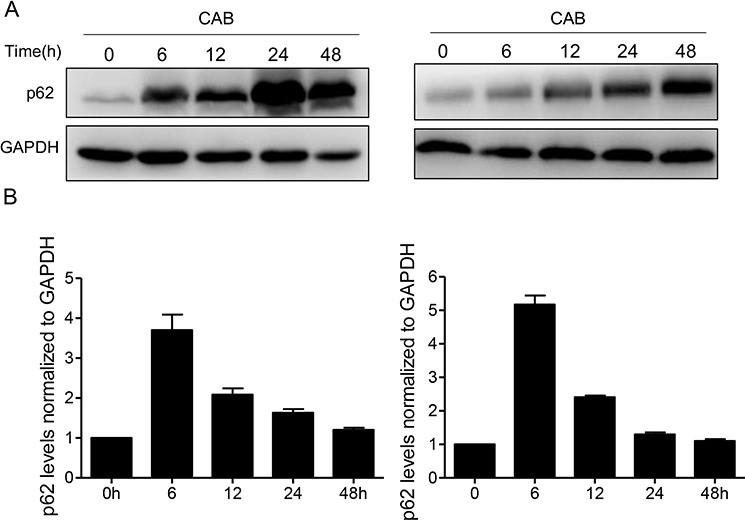
CAB blocks autophagic flux **A.** Immunoblotof p62 in MMQ (left panel) or GH3 (right panel) cells with or without CAB treatment at different time points. **B.** Analysis of p62 mRNA levels in MMQ (left panel) or GH3 (right panel) cells with or without CAB treatment at different time points.

Next, the integrity of autophagic flux was evaluated, because autophagy relies on lysosomes for the degradation of autophagosomal cargos. We checked whether CAB could induce the degradation of autophagy cargo p62, a marker of autophagic flux. Accumulation of p62 has been used as a marker of autophagy inhibition or indicator for defects in autophagic degradation [27, 28]. Using Western blotting, we observed a time-dependent increase in p62 levels after the CAB treatment in the MMQ and GH3 cells (Fig. 4A). The increase in p62 was evident as early as 6 h after initial CAB exposure in the MMQ cells. To rule out the possibility that the accumulation of p62 was due to increased p62 transcription, we measured the p62 mRNA level. We found that CAB promoted a small increase in p62 mRNA transcription after 6 h; however, the p62 mRNA level declined dramatically after 12 h of CAB treatment (Fig. 4B), indicating that the accumulation of p62 was not due to increased p62 transcription; but rather, degradation of the p62 protein was blocked and autophagic flux was disrupted in CAB-treated cells.

**Figure 5:**
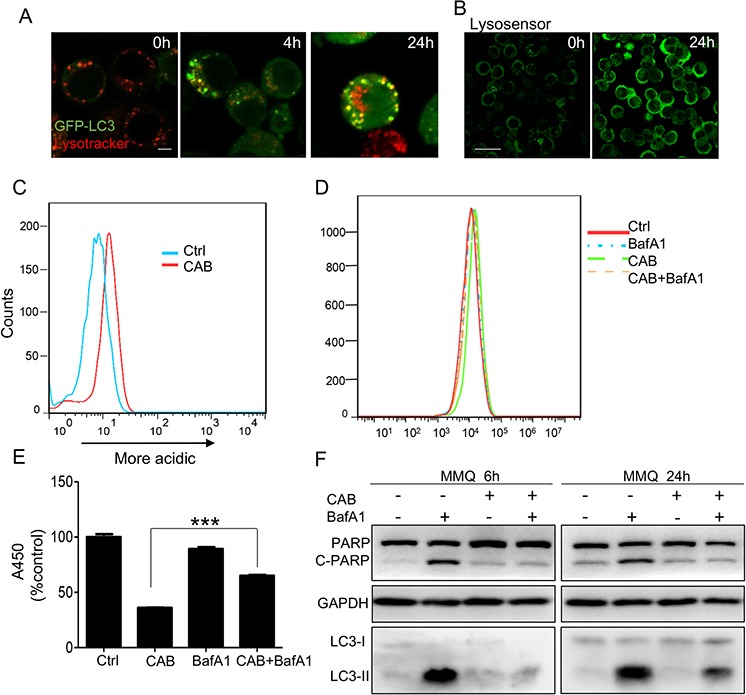
CAB impairs lysosomal degradation within autolysosomes **A.** CAB-treated MMQ (GFP-LC3) cells stained with Lysotracker. Scale bar: 5 μm. **B.** Following CAB treatment for 24 h, cells were loaded with 1 μM Lysosensor Green and examined with confocal microscopy. Scale bar: 20 μm. **C.** Samples from B were analyzed by flow cytometry. **D.** MMQ cells treated with DMSO, CAB, BafA1, or CAB+BafA1were loaded with 1 μM Lysosensor Green for 24 h and analyzed by flow cytometry. **E.** MMQ cells treated with DMSO, CAB, BafA1, or CAB+BafA1 assayed for cell proliferation in 24 h. **F.** Immunoblots analysis of PARP in MMQ cells treated with DMSO, CAB, BafA1, or CAB+BafA1 for 6 h and 24 h. ***p < 0.001.

To determine the reason for the disruption of the autophagic flux, we first ruled out the possibility of fusion dysfunction between the autophagosomes and the lysosomes. TEM clearly demonstrated the accumulation of autolysosomes inside the MMQ cells, indicating intact fusion function between the autophagosomes and the lysosomes (Fig. 1G and Fig. 3C). In addition, we found that after 4 h CAB treatment, MMQ cells showed a punctate pattern of GFP-LC3 fluorescence, while after 24 h CAB stimulation, GFP-LC3 and lysotracker largely colocalized, indicating the formation of LC3 and the intact fusion between autophagosomes and the lysosomes (Fig. 5A). Staining with lysosensor green DND-189, a pH indicator that exhibit a pH-dependent increasein fluorescence intensity upon acidification, revealed that the autolysosomes in MMQ cells after 24 h of CAB treatment exhibited much higher fluorescence intensity than that in control cells (Fig. 5B). Flow cytometry further demonstrated that CAB markedly augmented lysosome acidification (Fig. 5C).

BafA1, a specific inhibitor of vacuolar-type H (+)-ATPases, increased the lysosomal pH and reversed the CAB-mediated lysosomal acidification (Fig. 5D). Moreover, BafA1 consistently reversed the CAB-induced decline in cell viability (Fig. 5E) and inhibited the CAB-induced LC3-II accumulation and PARP expression at 6 h and 24 h, respectively (Fig. 5F). Those findings indicate that CAB augments lysosome acidification, leading to impaired lysosomal degradation and the accumulation of p62.

Taken together, our observations indicate that in sequential events, CAB first triggers autophagy but then progressively disrupts the autophagic flux, leading to an accumulation of undigested autolysosomes.

### The modulation of autophagy alters CAB-induced cell death

**Figure 6:**
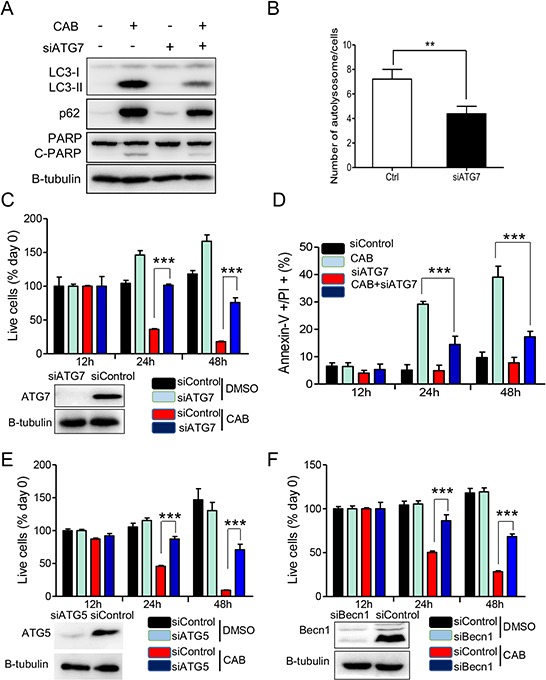
Silencing of autophagic key genes rescues CAB-induced cell death **A–D.** MMQ cells were transfected with control (Ctrl) or with ATG7 siRNA for three days before treating with CAB or DMSO, followed by immunoblot analysis for LC3, p62, PARP and β-tubulin (A) Histogram shows the average number of autolysosome seen in electron microscope (B), cell proliferation assay (C), and Annexin-V/PI staining assay (D). **E–F.** MMQ cells were transfected with control (Ctrl) or with ATG5 or Becn1 siRNA for three days before treating with CAB or DMSO, followed by cell proliferation assay. At the end of incubation, the cell survival rates were determined by CellTiter-Glo^®^luminescent cell viability assay and the cell survival rate was calculated. Results were reported as the mean ± SD of three independent experiments performed in four replicates. **p < 0.01, ***p < 0.001.

Over-activation of autophagy has been reported to induce ACD [29, 30]. To test whether increased autophagy and disrupted autophagic flux was responsible for cell death, we applied siRNA to effectively knockdown expressions of ATG7, which is required for autophagy initiation, and then tested viability of MMQ cells. Silencing of ATG7 decreased LC3-II levels and substantially reduced cleaved PARP (Fig. 6A), and also reduced the number of autolysosomes per cell (7.2 ± 0.8 vs 4.3 ± 0.6, *n* = 50, *p* = 0.0072, Fig. 6B). Knockdown of ATG7 almost completely reversed CAB-mediated decrease of MMQ cell viability at 24 hours (Fig. 6C). At 24 h, cell death was decreased by 22% after silencing of ATG7 (from 39% to 17%, shown in Fig. 6D). Furthermore, knockdown of Becn1 and ATG5, two other autophagy essential genes, suppressed CAB-induced cell death as well (Fig. 6E and 6F). Similar to autophagy inhibition by siRNA, the chemical inhibition of autophagy by 3-MA also reversed CAB-mediated cell death in MMQ cells (Supplementary Fig. 2A). Similar results were observed in GH3 cells, too (Supplementary Fig. 3A and 3B).

Together, these findings indicate that induction of autophagy by CAB is required for cell death.

### CAB inhibits tumor growth in MMQ xenograft model

**Figure 7:**
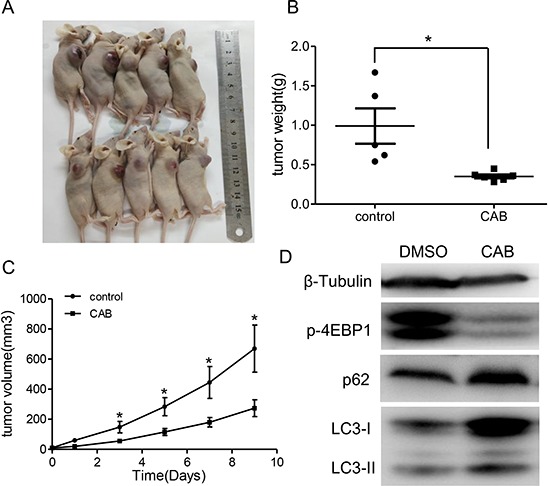
The growth-suppressive effect of CAB on MMQ cells *in vivo* **A–C.** CAB treatment inhibited the *in vivo* tumor growth of MMQ cells. The representative images for xenograft tumor on the nude mouse are shown in Figure 7A, and the tumor weight and tumor growth curve is shown in Figure 7B and 7C, respectively (*n* = 5). **D.** Immunoblot analyses showing p-4EBP1, p62, LC3 and β-tubulin in tumor samples. The protein on behalf of the average of the five tumor tissues.

Finally, we investigated whether CAB suppressed *in vivo* tumor growth in a MMQ xenograft model. Nude mice with MMQ xenograft were divided into control group and CAB treatment group. We found that the tumor sizes and the weights in the CAB treatment group were significantly smaller when compared with that of the control group (273.7 ± 55.5 mm^3^ in CAB treated group vs. 669.4 ± 156.7 mm^3^ in the control group, *P* = 0.029; Fig. 7A, 7B and 7C). Tumor weight was 0.35 ± 0.02 gand 0.99 ± 0.22 g in the CAB treatment group and control group, respectively (*P* = 0.01). Consistently, substantial up-regulation of LC3-II and p62 protein, and down-regulation of p-4EBP1 were observed in tumors from CAB treated group (Fig. 7D).

These findings show that CAB suppresses *in vivo* tumor growth by induction of autophagy and blockade of autophagy flux.

## DISCUSSION

We demonstrate for the first time that CAB induces autophagy-dependent cell death as evidenced by [31]: (1) CAB induces autophagy as indicated by the conversion of LC3-I to LC3-II, increased GFP-LC3, and autophagosome formation; (2) CAB blocks autophagic flux, leading to impaired or retarded autophagic degradation and substantial accumulation of autophagosomal cargos such as p62; (3) knockdown of ATG7, ATG5, and BECN1 can reduce CAB-mediated cell death. These findings elucidate a novel mechanism for CAB treatment of prolactinomas, and suggest its potential therapeutic use for other tumors.

CAB converts autophagy into a cell death mechanism by concomitant induction of autophagy and inhibition of the autophagic flux. Treatment of CAB in MMQ and GH3 cells resulted in rapid inhibition of mTOR activity and striking induction of LC3-II levels, along with a marked increase in the number and size of GFP-LC3-positive autophagosomes, suggesting induction of autophagy [32]. On the other hand, CAB blocks autophagic flux, causing accumulation of p62 and undigested autophagosomes in which autophagic degradation was impaired or retarded [33]. The optimal lysosome pH is essential for lysosomal proteases to regulate degradation; at either lower or higher pH, lysosome protease activity is impaired [34]. CAB dramatically increases lysosome acidity, thus generated an unfavorable environment for proteases and resulted in impaired lysosomal degradation within autolysosomes, leading to blockade of autophagic flux. Following the blockade of autophagic flux, CAB converts autophagy into a cell death mechanism which was confirmed by knock-down of proteins essential for autophagy. Knock-down of ATG7, ATG5, and Becn1, as well as chemical inhibition of autophagy, can significantly decrease the CAB-mediated MMQ cell death, indicating that autophagy indeed played an important role in the cytotoxicity of CAB. It has been shown that genetic inhibition of the ATG genes can prevent autophagic cell death in human U937 cells [35] and in Bax^−/−^Bak^−/−^embryonic fibroblasts [36], indicating that inhibition of apoptotic machinery might unravel an alternative cell death pathway that involves autophagy. A number of reports have also confirmed inhibition of cell death by knockdown of ATG genes, demonstrating that autophagy plays an essential death-promoting role [37–40].

In MMQ cell, CAB-induced cell death involves both apoptotic and autophagic pathway. Our TEM results show the morphology of dying cells is accompanied by large-scale AVs of the cytoplasm, which is rarely seen in typically apoptosis. Apoptosis and autophagy are two parallel pathways leading to cell death; each is activated depending on the stimulus and cell type, and sometimes may overlapping with each other [41]. It has been shown that cytotoxic signals can induce autophagy in cells that are resistant to apoptosis [42], such as those expressing high Bcl-2 or Bcl-X_L_, or those being exposed to pan-caspase inhibitors, such as Z-VAD-FMK [35]. Also, autophagy may contribute to cytotoxic and apoptosis response [43]. In addition, autophagy may directly induce cell death, known as ACD [44]. We showed that MMQ cells showed Annexin-V staining and activation of caspase-3, which are features of typical apoptosis; but pan-caspase inhibitor Z-VAD-FMK only partially reduced cell death. It is possible that Z-VAD-FMK can also block other enzymes at the concentration used in this study, such as cathepsins, which have previously been reported to be involved in caspase-dependent and –independent cell death [45, 46]. Caspase-3 activation is not only a marker of apoptosis, but also immediately precedes autophagic cell death as well as ATG gene transcription [47]. Therefore, apoptotic and autophagic modes of cell death can coexist as consequence of intracytoplamsic release of lysosomal enzymes upon CAB treatment. Together, they are parallel pathway both contribute to cell death, as has been shown before [44, 48].

In summary, this study demonstrates that CAB upregulates the autophagy response through inhibiting mTOR pathways, and also augments lysosome acidification resulting in impaired lysosomal degradation within autolysosomes, which severely blocks the autophagic flux and leads to accumulation of undigested cargos. Persistence of autophagy blockade disrupts the balance between autophagosome production and appropriate degradation capacity, which ultimately results in ACD. These findings present new insights into our understanding of mechanisms of CAB treatment for prolactinomas, as well as provide a novel therapeutic strategy for the medical management of other tumors.

## MATERIALS AND METHODS

### Reagents and antibodies

Reagents were purchased as follows: Cabergoline (Catalog #2664, Tocris, Bristol, UK); anti-phospho-p70s6k (Catalogue #9208), anti-p70s6k (Catalogue #2708), anti-phospho-4EBP1 (Catalogue #2855), anti-4EBP1 (Catalogue #9644), anti-phospho-mTOR (Catalogue #5536), anti-mTOR (Catalogue #2983) and PARP (Catalogue #9542) were from Cell Signaling Technology (Danvers, MA); Anti-β-tubulin (Catalogue #ab151318), Anti-GAPDH (Catalogue #ab181602), anti-ATG7 (Catalogue #ab133528), and anti-p62 (Catalogue #ab56416) were from Abcam (Cambridge, MA); LC3 (Catalogue #L7543) was from Sigma-Aldrich (St. Louis, MO); Becn1 (Catalogue #PD017) was from MBL (Woburn, MA). All antibodies were used according to manufacturers' instructions.

### Cell culture

Rat pituitary cell lines GH3 and MMQ were purchased from the American Type Culture Collection and cultured in DMEM/F12 medium (Gibco, Life Technologies, Grand Island, NY) supplemented with 2.5% fetal bovine serum (Gibco), 15% horse serum (Gibco) and 100 U/ml penicillin/streptomycin (Gibco).

### Cell viability and cell death measurement

Cell viability was measured using the MTS-based Cell Titer 96^®^AQueous One solution cell proliferation assay (Promega, Madison, WI) or CellTiter-Glo^®^ luminescent cell viability assay (Promega) according to the manufacturer's instructions. Upon addition of MTS solution, the reaction plate was incubated at 37°C for 3 h, and the absorbance was read at 490 nm with a plate reader (TECAN, Männedorf, Switzerland). For phosphatidylserine exposure, cells were stained with annexin V-PE as described by the manufacturer (BD Biosciences, San Jose, CA), and assayed by flow cytometry (CyAn ADP, Beckman Coulter, Brea, CA, USA).

### Expression constructs and transfection

MMQ and GH3 cells were transfected with GFP-LC3-II lentivirus from Genepharma, Shanghai. The siRNAs against rat ATG5, ATG7, and Becn1 were from Genepharma. The sequences of these siRNAs are:

siATG5: 5′ GCAUUAUCCAAUUGGCCUATT 3′
    5′ UAGGCCAAUUGGAUAAUGCTT 3′
siATG7: 5′ CAGCCUGGCAUUUGAUAAATT 3′
    5′ UUUAUCAAAUGCCAGGCUGTT 3′
siBecn1: 5′ CUCAGGAGAGGAGCCAUUUTT 3′
    5′ AAAUGGCUCCUCUCCUGAGTT 3′
siControl: 5′ UUCUCCGAACGUGUCACGUTT 3′
    5′ ACGUGACACGUUCGGAGAATT 3′

Cells were transfected using Lipofectamine RNAiMAX (Invitrogen, Carlsbad, CA, USA) according to the manufacturer's protocol.

### Real-time RT-PCR

Total RNA was extracted from MMQ and GH3cell lines using the Trizol reagent (Invitrogen), according to the manufacturer's instructions. The first-strand cDNAs were synthesized using a High-Capacity cDNA Archive Kit (ABI, Foster City, USA). Each cDNA (2 μl) was amplified in a SYBR Green Realtime PCR Master Mix (ABI) and loaded on the Applied Biosystems 7900 Real-time PCR Detection System (ABI). Thermal cycling conditions were as follows: 95°C for 10 min followed by 40 cycles of 95°C for 15 s, 60°C for 60 s, and 72°C for 30 s. PCR primers used were as follows: p62 (Rat-Forward): 5′-GACCCCACTTGAGATTCGT-3′; p62 (Rat-Reverse):5′-TGCTCCATCAGAGGATCCCA-3′; GAPDH(Rat-Forward): 5′-ACCCTGTTGCTGTAGCCATATTC-3′; GAPDH(Rat-Reverse): 5′-ACCCTGTTGCTGTAGCCATATTC-3′.

### Western blotting

Cell extracts for Western blotting were prepared in RIPA buffer (1 × PBS, 1% Nonidet P-40, 0.5% sodium deoxycholate, 0.1% SDS, 1 mM phenyl methylsulfonyl fluoride, and protease inhibitors). Lysates were separated by SDS-PAGE and were transferred to PVDF membranes (Millipore, Billerica, MA); the immune complex was detected by chemiluminescence (GE Healthcare, Wauwatosa, WI).

### Transmission electron microscopy (TEM)

Samples were processed in the Electron Microscopy Core at Fudan Univeristy. Cell pellets were fixed with 2.5% glutaraldehyde in 0.1 M phosphate buffer. The cells were washed with 0.1 M sodium cacodylate buffer and postfixed with 1% osmium tetroxide. The pellets were then dehydrated in graded ethanol series, infiltrated, and embedded in Spurr's resin. Samples were then polymerized for 48 h at 60°C, cut into 60-nm-thick sections on LKB-I microtome, positioned on 200 mesh grids, and stained with uranyl acetate and lead citrate. TEM was performed on PHILIPS CM120 TEM at an accelerating voltage of 120 Kv. Images were acquired with Gatan type UltraScan 4000SP CCD Camera connected to the TEM.

### Staining

For staining of lysosensor green DND-189, a pH indicator that exhibits a pH-dependent increase in fluorescence intensity upon acidification, cells were labeled with 1 μM LysoSensor Green DND-189 (Invitrogen) for 30 min. After the probe-containing medium was replaced with fresh medium, the cells were examined under a fluorescence microscope. For Lysotracker staining, cells were labeled with 0.5 μM lysotracker (Invitrogen) for 10 min, and then the probe-containing medium was replaced with fresh medium.

### Tumor formation assay

Five-week-old female athymic nude mice were purchased from the SLAC (Shanghai, China). One million MMQ cells in PBS were subcutaneously injected into the right back of each nude mouse. The animals were assigned randomly to two groups and the tumors were allowed to grow to ~50 mm^3^ in size. At this point, vehicle or CAB (0.5 mg/kg/d) in 100 μl of 0.9% saline was administered daily by gavage. Tumor volumes was measured by a vernier caliper every day and calculated as (length × width^2^)/2. Ten days later, all mice were sacrificed and tumors were harvested, followed by photography and Western blot. All procedures were performed in accordance with the National Institutes of Health Guide for the Care and Use of Laboratory Animals.

### Statistical analysis

Experimental results were analyzed with a Student's *t* test and graphed using Prism software (GraphPad Software, Inc., La Jolla, CA). Data are expressed as mean ± SD. A *p* value < 0.05 was considered statistically significant.

## SUPPLEMENTARY FIGURES



## Abbreviations

DA: Dopamine agonist
BRC: bromocriptine
CAB: cabergoline
PRL: prolactin
D2R: Dopamine 2 receptor
D2S: D2R short isoform
ACD: autophagic cell death
LC3-I: microtubule-associated protein light chain 3-I
LC3-II: microtubule-associated protein light chain 3-II
TEM: transmission electron microscopy
BafA1: Bafilomycin A1
GFP: green fluorescent protein
mTOR: mammalian target of rapamycin
4EBP: elongation factor 4E binding protein
p70S6K: p70 ribosomal protein S6 kinase
PARP: Poly (ADP-ribose) polymerase
AV: autophagic vacuole
p62: sequestosome-1
