# Supplementary material for: Suppression of mTOR pathway and induction of autophagy-dependent cell death by cabergoline
Source: Oncotarget. 2015 Oct 14;6(36):39329–41. doi: 10.18632/oncotarget.5744 (PMC4770775; doi:10.18632/oncotarget.5744)
Supplement: Supplementary file 1 [file oncotarget-06-39329-s001.pdf]

SUPPLEMENTARY FIGURES

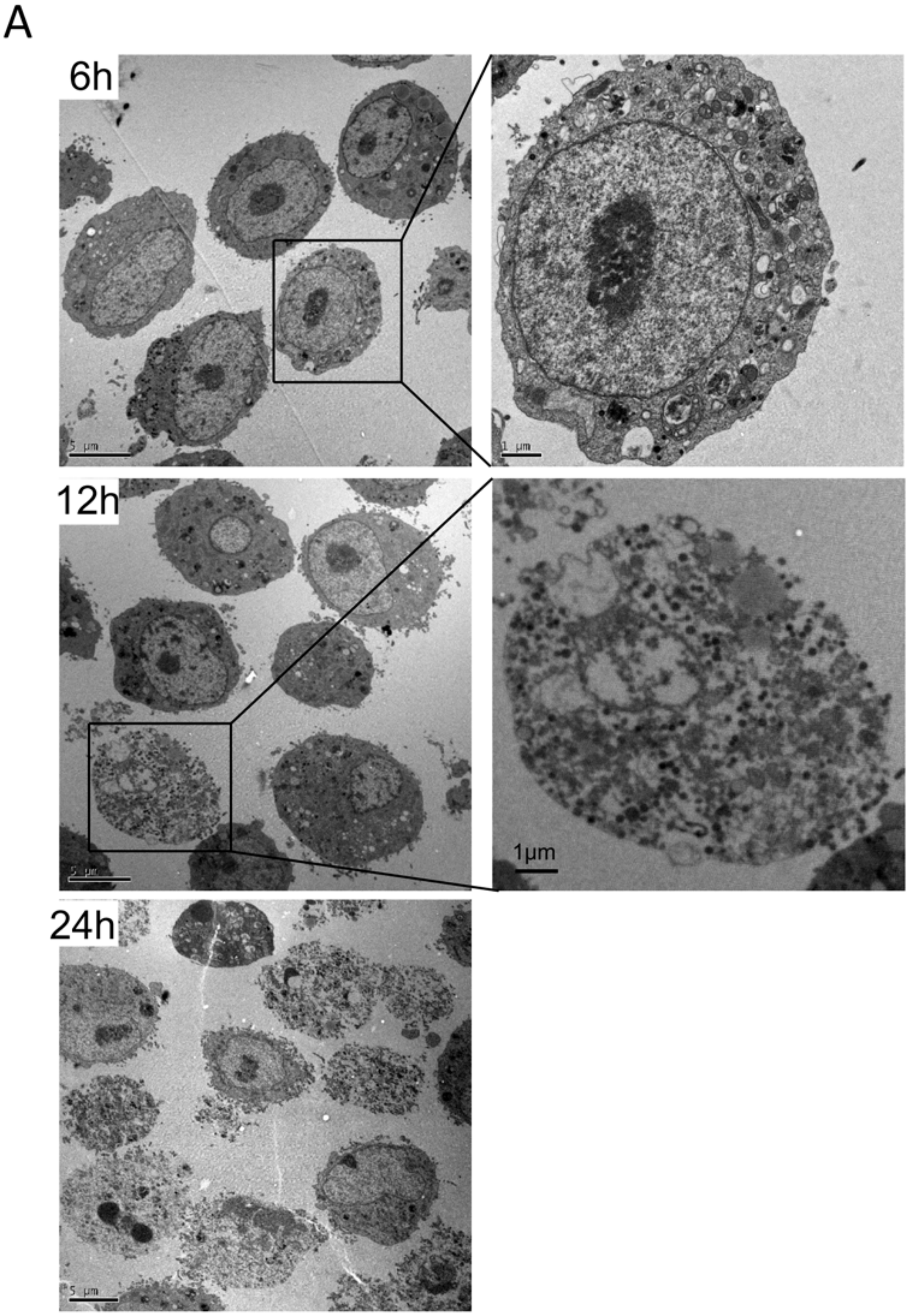

Supplementary Figure S1: A. Transmission electron microscopy (TEM) images with enlargements (boxes) showed autophagic vacuoles observed in CAB-treated MMQ cells for 6, 12 and 24 h.

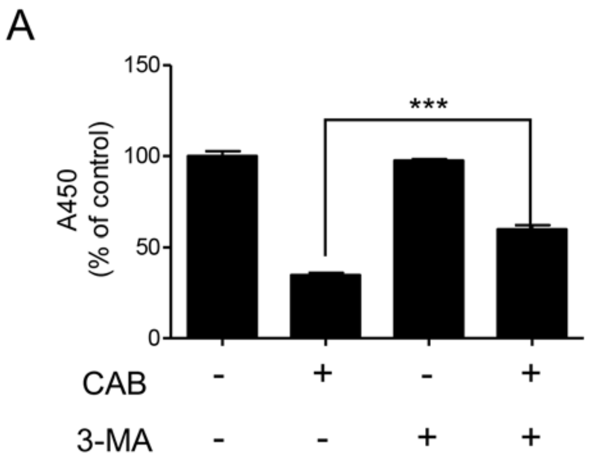

Supplementary Figure S2: A. MMQ cells were treated with CAB in the presence or absence of 3-MA for 48 h, and cell survival was determined using the MTS.

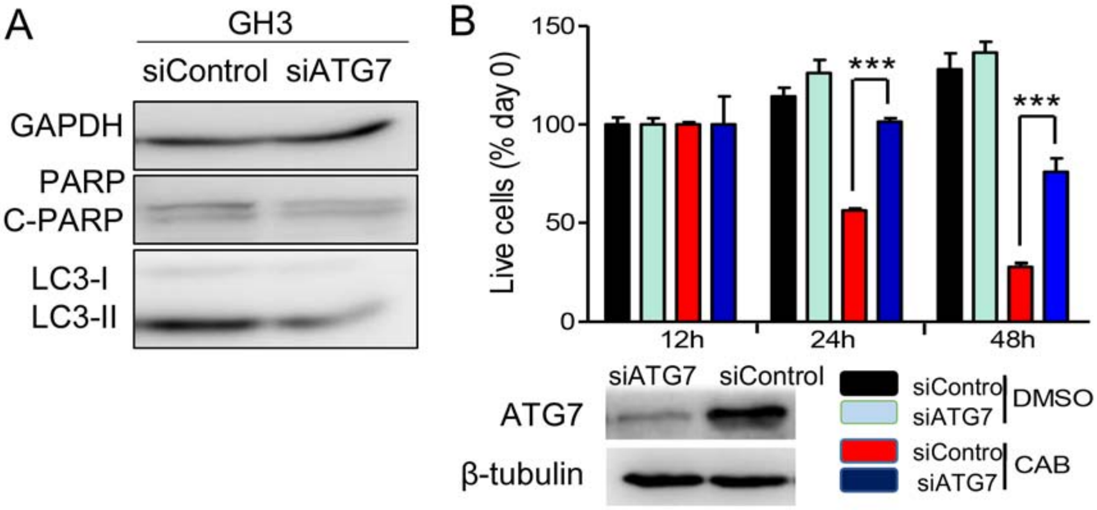

Supplementary Figure S3: GH3 cells were transfected with control or with ATG7 siRNA for three days before treated with CAB or DMSO for additional 3 days, followed by immunoblot analysis of LC3 (A) and cell proliferation assay (B).
